# Extensive diversity in the allelic frequency of *Plasmodium falciparum* merozoite surface proteins and glutamate-rich protein in rural and urban settings of southwestern Nigeria

**DOI:** 10.1186/s12936-017-2149-5

**Published:** 2018-01-02

**Authors:** Roland I. Funwei, Bolaji N. Thomas, Catherine O. Falade, Olusola Ojurongbe

**Affiliations:** 1 Department of Pharmacology and Therapeutics, College of Medicine, University of Ibadan, Ibadan, Nigeria; 2 Department of Pharmacy Technician Studies, Bayelsa State College of Health Technology, Yenagoa, Nigeria; 3 Department of Biomedical Sciences, College of Health Sciences and Technology, Rochester Institute of Technology, Rochester, NY USA; 4 Tropical Disease Research Laboratory, College of Health Sciences, Ladoke Akintola University of Technology, Osogbo, Nigeria; 5 Institute for Advanced Medical Research and Training, University of Ibadan, Ibadan, Nigeria; 6 Department of Medical Microbiology and Parasitology, Ladoke Akintola University of Technology, Osogbo, Nigeria

**Keywords:** Polymorphism, Genetic diversity, Merozoite surface protein, Glutamate rich protein, *Plasmodium falciparum*, Nigeria

## Abstract

**Background:**

Nigeria carries a high burden of malaria which makes continuous surveillance for current information on genetic diversity imperative. In this study, the merozoite surface proteins (*msp*-*1, msp*-*2*) and glutamate-rich protein (*glurp*) of *Plasmodium falciparum* collected from two communities representing rural and urban settings in Ibadan, southwestern Nigeria were analysed.

**Methods:**

A total of 511 febrile children, aged 3–59 months, whose parents/guardians provided informed consent, were recruited into the study. Capillary blood was obtained for malaria rapid diagnostic test, thick blood smears for parasite count and blood spots on filter paper for molecular analysis.

**Results:**

Three-hundred and nine samples were successfully genotyped for *msp*-*1*, *msp*-*2* and *glurp* genes. The allelic distribution of the three genes was not significantly different in the rural and urban communities. R033 and 3D7 were the most prevalent alleles in both rural and urban communities for *msp*-*1* and *msp*-*2*, respectively. Eleven of *glurp* RII region genotypes, coded I–XII, with sizes ranging from 500 to 1100 base pairs were detected in the rural setting. Genotype XI (1000–1050 bp) had the highest prevalence of 41.5 and 38.5% in rural and urban settings, respectively. Overall, 82.1 and 70.0% of samples had multiclonal infection with *msp*-*1* gene resulting in a mean multiplicity of infection (MOI) of 2.8 and 2.6 for rural and urban samples, respectively. *Msp*-*1* and *msp*-*2* genes displayed higher levels of diversity and higher MOI rates than the *glurp* gene.

**Conclusion:**

Significant genetic diversity was observed between rural and urban parasite populations in Ibadan, southwestern Nigeria. The results of this study show that malaria transmission intensity in these regions is still high. No significant difference was observed between rural and urban settings, except for a completely different *msp*-*1* allele, compared to previous reports, thereby confirming the changing face of malaria transmission in these communities. This study provides important baseline information required for monitoring the impact of malaria elimination efforts in this region and data points useful in revising current protocols.

## Background

Malaria caused by *Plasmodium falciparum* remains a major public health problem in most sub-Saharan African regions. Nigeria, one of the biggest nations by population in sub-Saharan Africa, accounts for the highest burden of the disease worldwide with about 30% under-5 years death, 25% infant death and 11% maternal mortality [1, 2]. The emergence and spread of drug-resistant *P. falciparum* in sub-Saharan Africa, the development of insecticide-resistant mosquitoes and failure to develop effective malarial vaccines has all added to the disease burden and eradication challenge [3]. Moreover, 60% of outpatient visits to hospitals is attributed to malaria, with the recent malaria-risk maps showing a prevalence that varied from 20 to over 70% [4]. The huge malaria burden in Nigeria, demands continuous understanding of the genotypic and phenotypic diversity of malaria parasites that enhances their ability to counteract control measures such as therapeutic drugs and insecticide treatment nets. In Nigeria, as in many other African countries, the dynamics and genetic composition of *P. falciparum* populations remain unclear. Dramatic variation in diversity and geographical differentiation have been reported in areas of different transmission intensities [5, 6]. Regions of low transmission showed low genetic diversity while random association among loci, high genetic diversity, and minimal geographical differentiation were observed in regions of Africa and Papua New Guinea, where transmission is intense implying a simple linear relationship between genetic diversity and transmission intensity [7]. Despite this clear trend of linearity, it has recently been shown in *P. vivax* that the relationship between diversity and transmission intensity is not simply linear because many patients could be infected by either related parasites resulting from inbreeding or distinct new parasites [8]. Also, the complexity of estimating genetic diversity may further be influenced by effective population size, the mutation rate of the loci under study and also the genotyping method being used [8]. Many studies have reported the extensive variations in the genotypic and phenotypic diversity of *P. falciparum*, a feature that has helped the parasite in overcoming anti-malarial drugs, vaccines and vector control strategies, but the dynamics and genetics of *P. falciparum* populations remain unclear. Moreover, geographic genetic differentiation exists which appear to be inconsistent, warranting the need to constantly assess the structure and diversity of *P. falciparum* in Africa.

The *P. falciparum* merozoite surface proteins 1 and 2 (*msp*-*1* and *msp*-*2)* and glutamate-rich protein (*glurp)* genes are significant molecular markers, highly effective in differentiating monoclonal from multiclonal infections within infecting parasite strains, including their utilization in discriminating recrudescence from re-infecting alleles [9, 10]. They are also parasite proteins under consideration as potential vaccine candidates, either individually or in combination with other parasite proteins, because of their preponderance at various stages of the parasite life cycle [11–14]. The *msp*-*1* and *2* are erythrocyte membrane-bound proteins abundantly found on the blood stages of *P. falciparum* infected erythrocytes, and reported to play major roles in erythrocyte invasion [11]. It is highly polymorphic with substantial polymorphisms across the protein particularly in *msp*-*1*-block 2 regions which has three allelic groups [11]. On the other hand *msp*-*2* is dimorphic, existing in two main allelic forms of 3D7-like and FC27-like family, sharing N- and C-terminal regions, with strain-specific variable regions. Merozoite surface protein 2 is suggested to be essential during parasite invasion and remains on the surface throughout invasion only to be degraded upon invasion completion [15]. The *glurp* gene is composed of a rather conserved region (R0 region), a central repeat region (R1 fragment) and a C-terminal immunodominant repeat region (R2 fragment) [16]. The polymorphic R2 region possesses at least two B cell epitopes that stimulate antibodies capable of inhibiting parasite growth in vitro [17]. The block 2 region of the *msp*-*1*, the central repetitive domain of the *msp*-*2* (block 3), and R2 region of the *glurp* gene have been well characterized in most malaria-endemic regions globally to study the parasite genetic structure and the allelic frequency distribution [10, 18, 19].

Since malaria is endemic in Nigeria, it is important to characterize these markers from time to time in order to elucidate the genotypic pattern of the circulating parasite populations, understand the mechanism underlying disease pathogenesis, transmission and acquisition of drug resistance in parasite strains with the intent of establishing an effective control strategy. The assemblage of baseline data for these polymorphic biomarkers in the parasite population from different geographical regions and different levels of malaria transmission in urban and rural areas are essential tools for the current efforts on malaria control strategies in sub-Saharan Africa [20, 21]. Significantly and probably more pertinent to this study are anecdotal reports stating that the patterns of infection and disease severity differs between rural and urban areas [22, 23]. If so, how would this affect control programmes or institution of national treatment guidelines between rural and urban areas among infected patients? Such strategies would require up-to-date surveillance information possible via molecular characterization of these genes in parasite samples recovered from patients presenting with acute *P. falciparum* infection from rural and urban areas. To this end, this study report the results of the genetic analysis of *msp*-*1, msp*-*2* and *glurp* genes among children under 5 years of age, presenting with acute uncomplicated *P. falciparum* infection, recruited from rural and urban areas of Nigeria.

## Methods

### Study site and patients

This was a cross-sectional study conducted in a primary healthcare facility at Idi-Ayunre (rural) and St Mary Catholic Hospital, Eleta (urban), Ibadan from November 2013 to November 2014. Idi-Ayunre is the headquarters of Oluyole Local Government Area, with an estimated area of 629 sq km and a population of approximately 202,725 based on 2006 population census [24]. St Mary’s Catholic Hospital, Eleta is a secondary healthcare facility located within the city of Ibadan, the Oyo State capital. Both study sites have tropical, wet (running from April through October) and dry (running from November to March) seasons, with malaria transmission occurring all year round [25]. A total of 511 children, aged 3–59 months (rural 365 *vs* urban 146), presenting with fever (axillary temperature ≥ 37.5 °C) or a history of fever in the past 48 h, and other inclusion criteria, were enrolled into the study. Using standard aseptic technique, capillary blood obtained from a fingerprick on the middle finger after cleaning with 70% alcohol was used to prepare thick blood smears and spotted on filter paper (Whartman 3MM™) for future molecular analysis. Thick blood smears were air dried and stained with 10% fresh Giemsa, following standard procedures, and parasite density calculated by counting the number of asexual parasites against 200 leukocytes in the same high power fields, assuming a leukocyte count of 8000 white cells/µl [26].

### DNA extraction and PCR analysis

Finger-prick blood spotted on Whatman™ 3MM filter paper, were dried in dust free area and subsequently placed in a zip-lock bag with silica gel to prevent DNA degradation. Dried blood spots were utilized for DNA isolation and PCR amplification. DNA extraction was performed with the QIAamp DNA Mini kit (QIAGEN Germany), according to manufacturer’s instructions with slight modification. Genomic DNA eluates were stored at − 20 °C until further PCR analysis.

### Amplification of *Plasmodium falciparum*

The small sub-unit ribosomal RNA (ssrRNA) gene of *P. falciparum* was amplified in a nested PCR using primary genus-specific primers and a secondary *P. falciparum* species-specific primers following the previously described standard protocol [27] (Table 1). Amplified genomic DNA samples confirmed for *P. falciparum* were further characterized for *msp*-*1, msp*-*2* and *glurp* genes, with specific oligonucleotides primer pairs.

**Table 1:** General characteristics of the study population

	Number examined n = 511 (%)	Number positive n = 309 (%)	*p* value
Study sites			
Rural	365 (100.0)	250 (68.5)	< 0.001*
Urban	146 (100.0)	59 (40.4)	
Sex			
Male, (%)	301 (100.0)	188 (62.5)	0.271
Female, (%)	210 (100.0)	121 (57.6)	
Age (months)			
Range	3–59	3–59	–
Mean	26.4 ± 15.7	29.4 ± 15.3	< 0.001*
Mean parasite density			
Rural (range) ± SD	–	2.67E4 (113E3–360E3) ± 52,099	0.383
Urban (range) ± SD	–	3.50E4 (76E3–331E3) ± 76,180	

* Significant p < 0.05

### Allelic genotyping of *Plasmodium falciparum msp*-*1, msp*-*2* and *glurp* genes

Primers specific for the polymorphic region of *msp*-*1* and *2* (block 2 and 3), as described previously [27], were used for genotyping. The polymorphic allelic families of *msp*-*1* (K1, MAD20, and RO33) and the block 3 region of *msp*-*2* (FC27 and 3D7) genes were amplified with a nested PCR amplification in a final reaction volume of 20 µl containing 10X PCR buffer (20 mM Tris–HCl [pH 8.4], 50 mM KCl, and 1.5 mM of MgCl2), 0.125 mM of dNTPs, 0.25 mM of each primer, and 1 U Taq DNA polymerase (New England Biolabs. Inc, UK) and 5 µl of DNA in the first reaction and 3 µl of the first-round product in the second (nested) reaction was added as template. The PCR cycling conditions for both primary and nested reactions are as previously described [28].

For the *glurp* gene, a semi-nested PCR reaction in a final volume of 25 µl for the polymorphic R2 region was performed with specific primers containing PCR reagent concentrations as in *msp*-*1* and *msp*-*2* genotyping above [27]. The cycling conditions were the same for both reactions except the annealing temperature for the second reaction was at 59 °C. The resultant PCR products were stained with SYBR^®^ Green 1 nucleic acid gel stain (Cambrex Biosciences, East Rutherford, NJ, USA) and resolved by gel electrophoresis in 1.5% agarose gel. DNA sizes were determined using 100 bp DNA ladder (New England Biolabs. Inc, UK), and photographed under UV trans-illuminator (UVP^®^ DigiDoc-It™, USA).

## Results

A total of 511 children were screened for malaria infection in a rural and an urban community in southwest, Nigeria. Three-hundred and nine enrollees (60.5%; mean age 26.4 ± 15.7 months; range 3–59) were confirmed positive by nested PCR for *P*. *falciparum* infection. Specifically, 68.5% (250/365) of the PCR confirmed *P. falciparum* cases was from the rural community while 40.4% (59/146) was from the urban community. The difference was statistically significant (p < 0.001) (Table 1). Mean parasite density was higher in the urban community compared to the rural community but the difference was not statically significant (p = 0.383).

Five different *msp-1* alleles were observed in this study, with RO33 being the most common allele in the rural community with a monoclonal infection frequency of 7.3%. The frequency of multiclonal infection (RO33 + MAD20 + K1) was 82.1%. The multiplicity of infection (MOI) for merozoite surface proteins-1 in the rural community was 2.8. For the urban community, both RO33 and MAD20 had a monoclonal infection frequency of 10.0% each, frequency of multiclonal infection (RO33 + MAD20 + K1) at 70.0% and an overall MOI of 2.6 (Table 2).

**Table 2:** Distribution of MSP-1 allelic family in rural and urban communities

	MSP-1 family	No. of samples positively amplified	Allelic size (bp)	Frequency (%)	MOI
Rural (n = 151)	RO33	11	150–250	7.3	2.8
	MAD20	5	150–200	3.3	
	K1	4	150–350	2.6	
	RO33 + MAD20	4		2.6	
	RO33 + K1	3		2.0	
	RO33 + MAD20 + K1	124		82.1	
Urban (n = 30)	RO33	3	150	10.0	2.6
	MAD20	3	150–250	10.0	
	RO33 + K1	3		10.0	
	RO33 + MAD20 + K1	21		70.0	

Numbers sampled cannot add up since a sample can amplify in more than 1 family

*MOI* multiplicity of infection, *bp* base pairs, *MSP-1* merozoite surface protein-1

For merozoite surface protein-2, a total of 15 different alleles (8 alleles in 3D7: 200–1000 bp and 7 alleles in FC27: 200–800 bp) were identified (Table 3). The 3D7 allele was the most common in the rural community (monoclonal frequency of 43.2%), while FC27 monoclonal allele was not observed. About two-thirds (68.3%) of the samples were multiclonally infected having both alleles (3D7 + FC27). In the urban setting on the other hand, both 3D7 and FC27 alleles had monoclonal infection rates of 15% each and a multiclonal infection (3D7 + FC27) frequency of 70.0% each. The MOI value for *msp*-*2* genotype for rural and urban community was 2.1 and 1.9, respectively (Table 3).

**Table 3:** Distribution of MSP-2 allelic family in rural and urban communities

	MSP-2 family	No. of samples positively amplified	Allelic size (bp)	Frequency (%)	MOI
Rural (n = 88)	3D7	38	200–900	43.20	2.1
	3D7 + FC27	60		68.30	
Urban (n = 20)	3D7	3	300–600	15.00	1.9
	FC27	3	400–500	15.00	
	3D7 + FC27	14		70.00	

Numbers sampled cannot add up since a sample can amplify in more than 1 family

*MOI* multiplicity of infection, *bp* base pairs, *MSP-2* merozoite surface protein-2

Eleven *glurp* genotypes were detected in the rural community with size ranging from 500 to 1100 bp (50 bp bin) and coded as genotype I–XII with genotype XI the most predominant (41.5%). In the urban setting however, fewer genotypes were recorded with only six genotypes (II, VII, IX, X, XI, XII) detected. Genotype XI (1000–1050 bp) was also the most common. Genotype VI (750–800 bp) did not appear in both rural and urban communities and the MOI in both communities was 1.0 (Table 4).

**Table 4:** Distribution of allelic variants of GLURP RII repeat region of *P. falciparum* in rural and urban communities

Genotypes	Allelic size (bp)	Rural n = 106 (%)	Urban n = 26 (%)
I	500–550	1 (0.9%)	0 (0.0%)
II	550–600	2 (1.9%)	1 (3.8%)
III	600–650	2 (1.9%)	0 (0.0%)
IV	650–700	1 (0.9%)	0 (0.0%)
V	700–750	2 (1.9%)	0 (0.0%)
VI	750–800	0 (0.0%)	0 (0.0%)
VII	800–850	15 (12.2%)	2 (7.7%)
VIII	850–900	6 (5.7%)	0 (0.0%)
IX	900–950	17 (16.0%)	5 (19.2%)
X	950–1000	7 (6.6%)	3 (11.5%)
XI	1000–1050	44 (41.5%)	10 (38.5%)
XII	1050–1100	3 (2.8%)	3 (11.5%)
X + XI		2 (1.9%)	0 (0.0%)
XI + XII		4 (3.8%)	1(3.8%)
III + XI		0 (0.0%)	1 (3.8%)
MOI		1.0	1.0

*GLURP* glutamate rich protein

When parasite density was compared with prevalence of the different allelic families at parasite density ≥ 2000 parasites/µl, the K1, MAD20 and RO33 allelic families were amplified in 87.6% (85/97), 90.7% (88/97) and 94.8% (92/97) of positive samples, respectively. Similarly, FC27 was positive for 71.4% (65/91) while 3D7 accounted for 86.8% (79/91). At parasite density ≤ 2000 parasites/µl however, there was a reduction in the allelic frequencies, but not statistically significant, as K1, MAD20 and RO33 had 84.3% (70/83), 83.1% (69/83) and 91.6% (76/83), respectively. For *msp*-*2*, FC27 amplified for 68.2% (30/44) while 3D7 recorded 81.8% (36/44), respectively.

The allelic frequency for *glurp* gene was 100% (64/64) positive at parasite density ≥ 2000 parasites/µl, and 97.1% (66/68) at parasite density ≤ 2000 parasites/µl blood, an MOI of 1.1 (Table 5). There was no statistically significant difference in the prevalence of allelic families in relation to parasite density (*p* > 0.05).

**Table 5:** Distribution of MSP-1, MSP-2 and GLURP alleles by parasite density among children

Genes	Parasite count > 2000	Fragment size (bp)	MOI	Parasite count < 2000	Fragment size (bp)	MOI	*p*-value
*MSP1*	*n* *=* *82* (%)		2.6	*n* *=* *99* (*%*)		2.8	
R033	9 (11.0)	150–250		5 (5.1)	150–250		NS
MAD20	4 (4.9)	150–200		4 (4.0)	150–300		NS
K1	3 (3.7)	150–300		1 (1.0)	350		NS
RO33 + MAD20	1 (1.2)			2 (2.0)			NS
RO33 + K1	3 (3.7)			3 (3.0)			NS
MAD20 + K1	1 (1.2)			0 (0.0)			NS
RO33 + MAD20 + K1	61 (74.4)			84 (84.8)			NS
*MSP2*	*n* *=* *43 (%)*		1.8	*n* *=* *92 (%)*		2.0	
FC27	8 (18.6)	200–600		12 (13.0)	300–700		NS
3D7	15 (34.9)	300–900		26 (28.3)	200–600		NS
FC27 + 3D7	20 (46.5)			54 (58.7)			NS
*GLURP*	*n* *=* *66*		1.1	*n* *=* *66*		1.0	
	64 (97.0)	500–1050		66 (100.0)	500–1050		NS

## Discussion

This study evaluated the genetic diversity of *P. falciparum* polymorphic markers *(msp*-*1, msp*-*2* and *glurp* genes) in children from urban and rural communities of Oyo State, southwest Nigeria. Understanding the genetic diversity of *P. falciparum* in different geographical settings is of critical importance in resolving the population structure of the parasites in endemic areas and in order to develop effective control strategies [29, 30]. On the other hand, the extensive genetic diversity and complexity of *P. falciparum* is also considered a major obstacle in the acquisition of natural immunity and vaccine development efforts. The findings in this study are consistent with previous reports that showed that these genes are highly polymorphic among the parasite population in southwestern Nigeria [12, 31, 32]. The genetic diversity analysis in the present study showed that *P. falciparum* populations in Idi Ayunre (rural) and Eleta (urban) exhibited an overall similar level of high genetic diversity, with MOI of 2.8 and 2.6, respectively.

In the *msp*-*1* gene, RO33 was the most predominant allelic family in both rural (7.3%) and urban (10.0%) settings, contrary to previous reports by others from the same region where K1 allelic family was reported as the commonest [31, 33]. Incidentally, K1 was the least encountered genotype in both our study areas based on the findings of the study. Although the previous results were from similar study area and in patients of same ethnicity, the small sample size (n = 50 and 100, respectively), and years since study (13 years), grants significant credence to the present result and clarifies its uniqueness, revealing that the different predominant allele recorded illustrates the changing parasite profile. In addition, the higher MOI recorded in this study depicts the complexity of the circulating parasite population. This changing genetic make-up of *P. falciparum* parasite in this study may be due to high transmission intensity and the possible impact of different control measures, mainly chemotherapy and insecticide-treated nets (ITNs). Change in genetic make-up of *P. falciparum* has been reported in endemic communities following change in anti-malarial drugs and introduction of ITNs [34]. The findings of this study are consistent with the high proportion of RO33 allelic family reported in Senegal [35], Sudan [36], Uganda [37], and Thai-Myanmar borders [18], which were associated with high malaria transmission and a decline in response to treatment with artesunate-mefloquine. For *msp*-*2* locus, the 3D7 allelic family was the most abundant single allele in both communities. This is similar to that reported from the Thai-Myanmar border [18], Nigeria [38], Congo Brazzaville [10], as well as Peru and Iran [39, 40], but different from previous results from Osogbo, Nigeria [19] and northeastern Myanmar [41]. Differential distribution of *msp* alleles has been associated with clinical status yielding conflicting reports. Some studies have reported that RO33 and FC27 alleles are typically frequently observed in asymptomatic malarial infections [42], others have reported this not to be [38, 43, 44].

High prevalence of multiclonal infection was observed with *msp*-*1* and *msp*-*2* genotypes in both community settings. This persistent high level of genetic diversity is an indication that the parasite population size remained large enough to allow effective mixing of genotypes. There is also the possibility of human migration, which may also bring about increase and changes in genetic diversity by introducing additional parasite genotypes [45]. Significantly, the data from this study showed higher allelic sizes for *msp*-*1* variants (up to 350 bp) and similar *msp*-*2* sizes viz *a* viz previous report [31], contributing to the changing picture of parasite diversity in southwest Nigeria. Of additional importance is the fact that the highest allelic size reported (350 bp) was found in the rural setting, potentially indicating a higher rate of recombination within parasite species in rural than urban areas.

The RII region of *glurp* gene also demonstrated a high degree of polymorphism in the parasite population, with 12 distinct allelic fragment sizes, out of which 11 were observed in the rural setting and six observed in the urban community. The high number of *glurp* alleles in the study area is not unexpected, considering the high malaria endemicity, which is also in agreement with previous reports [10, 46]. The low number of *glurp* alleles observed in the urban area in comparison to the rural area could also be an indication of higher transmission intensity in the rural area, including significant changes in the parasite genome happening at a faster pace in the rural than urban areas.

Interestingly, when parasite density was categorized into two groups (> 2000 and < 2000 parasites/µl), the genetic diversity was not influenced by parasite density. This is indicative of the fact that high malaria transmission and parasitaemia may not have strong association with genetic diversity of *P. falciparum* in the study areas. The association of *P. falciparum* genetic diversity on clinical malaria (indicated as parasitaemia in this study) has been yielding conflicting report. Previous work failed to establish a significant association between different allelic families of *msp*-*2* and parasite density [19], with the reverse reported from Central Sudan [36] and Congo Brazzaville, [47], showing positive correlation between parasite density and the number of *msp*-*1* and *msp*-*2* genotypes. Additionally, previous association between the *msp*-*2* locus of *P. falciparum* and clinical severity of malaria in children, reported from Ibadan [38], was not replicated in this study. This is not unusual in hyper-endemic locations, with possible reasons including potential co-infection with different parasite variants or super-infections, intra-host dynamics such as overlap of parasite variants between primary and secondary infections, and differential degree of host immunity and exposure [48–50]. Continuous surveillance and monitoring of circulating parasite variants towards the design of effective control measures as well as effectiveness of current anti-malarial therapy in such locales is therefore recommended.

## Conclusion

Despite the ongoing extensive malaria control efforts in Nigeria, the prevalence of malaria remain high, especially in the rural communities and the *P. falciparum* parasite population remains highly diverse at the three antigenic loci (*msp*-*1*, *msp*-*2* and *glurp*) analysed in this study. The high MOI is suggestive of effective genetic recombination of the parasite population, which may be related to the high transmission of *P. falciparum* in the study area, leading to continued maintenance of genetic diversity. The data could serve as an important baseline information for understanding the population structure of *P. falciparum* and can be harnessed into malaria control strategies in an effort aimed at eliminating malaria in Nigeria.
